# How to meet the demand for good quality renal dialysis as part of universal health coverage in resource-limited settings?

**DOI:** 10.1186/s12961-016-0090-7

**Published:** 2016-03-18

**Authors:** Yot Teerawattananon, Alia Luz, Songyot Pilasant, Suteenoot Tangsathitkulchai, Sarocha Chootipongchaivat, Nattha Tritasavit, Inthira Yamabhai, Sripen Tantivess

**Affiliations:** Health Intervention and Technology Assessment Program (HITAP), Department of Health, Ministry of Public Health, Tiwanon road, Nonthaburi, 11000 Thailand

**Keywords:** Chronic kidney disease, End-stage renal disease, Low- and middle-income countries, Peritoneal dialysis, Renal replacement therapy, Universal health coverage

## Abstract

**Background:**

It is very challenging for resource-limited settings to introduce universal health coverage (UHC), particularly regarding the inclusion of high-cost renal dialysis as part of the UHC benefit package. This paper addresses three issues: (1) whether a setting commits to include renal dialysis in its UHC benefit package and if so, why and how; (2) how to ensure quality of renal dialysis services; and (3) how to improve the quality of life of patients using psychosocial and community interventions.

**Discussion:**

This article reviews experiences of renal dialysis programs in seven settings based on presentations and discussions during the International Forum on Peritoneal Dialysis as a Priority Health Policy in Asia. A literature review was conducted to verify and validate the data as well as to fill information gaps presented in the forum.

Five out of the seven settings implemented renal dialysis as part of their benefits package, while the other two have pilots or programs in their nascent stage. Renal replacement therapy has become part of the universal access package because these governments recognize the rising number of chronic kidney disease (CKD) cases, the catastrophically high costs of treatment, and that this is the only life-saving treatment available to patients. The recommendations are as follows: Governments should have a holistic approach to CKD interventions, including primary prevention as well as psychosocial interventions. Governments should consider subsidizing CKD treatment costs depending on their resources. Multi-stakeholder cooperation should be facilitated to enact these policies and conduct research and development for all aspects of interventions. International collaboration should be initiated to share experiences, good practices, and joint activities (e.g. capacity building and multinational procurement of medical supplies).

**Conclusion:**

This study provides practical recommendations to country governments as well as the international community on how to meet the demand for good quality renal dialysis as part of UHC in resource-limited settings.

## Background

During the last decade, many low- and middle-income countries (LMICs) declared major reforms in their health systems to achieve universal health coverage (UHC). Although UHC has been differently interpreted, defined and translated into action [1], the ultimate goal of such a policy as proposed by WHO is to promote population health by ensuring universal access to essential healthcare of high quality, protecting people from health impoverishment and improving equity in health across socioeconomic groups [2]. In so doing, country governments need to take into consideration three key dimensions, namely the population to be covered as beneficiaries, the services to be included in the benefit package, and the proportion of public subsidization for healthcare provision [3]. Following the World Health Assembly resolution on sustainable health financing for UHC, the policy options to be adopted should be pertinent to the economic, sociocultural and political context of each country [4].

Every country which moves towards UHC has to raise additional funds, either from domestic sources or from international development support [3]. It is evident that, in many LMICs, additional public resources have been mobilized in order to guarantee access to the necessary services for all people in need. For instance, in Thailand, where a UHC scheme was established in 2002, government spending on health has continued to grow, and a significant increase of 75% was observed between 2005 and 2010 [5]. However, the demand for a higher level of public health budget cannot be met in all countries, although innovative financing mechanisms have already been introduced. In particular, most resource-poor settings have been facing a double burden of infectious diseases and non-communicable diseases [6]. Some argue that increasing access to healthcare can yield substantial economic benefits in the macro and micro level, including protecting households from illness-related catastrophe [2]. However, since it is usually difficult to link economic growth with health investment, and since it takes a long time to see economic contribution of health initiatives, allocating a large amount of the already limited resources to UHC or even particularly expensive health interventions is politically challenging [7].

Renal replacement therapy (RRT), including renal dialysis and transplantation, for end-stage renal disease (ESRD) is a high-cost, albeit life-saving service. On a global level, it is estimated that 1 in every 20 people have chronic kidney disease (CKD). ESRD – the last and most critical stage of CKD – is a growing problem in most countries. Its incidence is increasing globally at an annual growth rate of 8% [8]. The 2014 United States Renal Data System report shows that the incidence of ESRD increased by 10% (per million population) from 2006 to 2012 in Korea, Malaysia, Mexico, Thailand, and the USA [9]. People over 65 years of age are more vulnerable to CKD than those in other age groups, and also have a higher probability of death [8]. The vascular and systemic complications in this subpopulation does not make them optimal candidates for renal transplantation, and they are less likely to receive a kidney from a living related donor. The costs for RRT are generally too high for most patients in developing countries to afford [10]. Furthermore, kidney donor and transplantation services are not widely available in resource-limited settings [11]. Liyanage et al. [12] estimated that, in 2010, around 2.3 million people died from lack of access to RRT, and worldwide use of RRT will double from 2.6 million to 5.4 million people by 2030. Providing treatment for ESRD patients is becoming more of a concern, especially since most treatment options are costly and would need to be balanced with provision of other health services.

Despite the notable budget implication of RRT, some countries have made strides towards alleviating the burdens for ESRD by subsidizing renal dialysis for their people, with or without co-payment from households. Besides the financial obstacle, inadequate infrastructure and trained personnel are major supply-side barriers to UHC of renal dialysis in resource-poor settings [10]. At the same time, access and adherence to long-term health services are hindered by factors of the demand side. For renal dialysis, these include effective coverage, quality and health outcomes of two major modalities, peritoneal dialysis (PD) and haemodialysis (HD), which are associated with living circumstances, employment and education of ESRD patients; support from family members and community networks; and contextual elements such as distance between patients’ residence and health facilities and convenience in transportation [13, 14]. This means that providing universal access to renal dialysis requires not only public finance, but also suitable, integrated measures to address all the impediments.

Based on presentations and discussion during the *International Forum on Peritoneal Dialysis as a Priority Health Policy in Asia*, held in November 2014 in Bangkok, this article reviews experiences of renal dialysis programs in seven settings, namely Hong Kong, Indonesia, Malaysia, the Philippines, Taiwan, Thailand, and the United Kingdom. It emphasizes policy response to the rising CKD burden, clinical innovations to ensure quality of care, and psychosocial support provided by paramedics, community, non-governmental organizations and patient networks in each country context. The information gathered from personal communication to the experts was part of the preparation of the workshop and was also taken into account. Additionally, a literature review was conducted to verify and validate as well as fill information gaps presented in the forum. This paper will provide guidance to LMICs committed to UHC on effective ways to respond to the challenging issue of providing treatment to ESRD patients. Table 1 illustrates country/setting profiles in relation to their UHC policy and burden of CKD.

**Table 1:** Country/setting profiles for health systems and renal disease profiles

Country/Setting	Pledged year of achieving Universal Health Coverage	Population (million) (2013) [56]	Gross Domestic Product (GDP) per capita (USD) (2013) [57]	Total health expenditure as % GDP (2012) [58]	Prevalence of diabetes, per million population (2014) [59]	Prevalence of end-stage renal disease, per million population (2012) [9, 34]
Hong Kong Special Administrative Region	1993 [60, 61]	7.2	38,123.5	5.1 [62]	99,200	1,192
Indonesia	2019 [63, 64]	249.9	3,475.3	3.0	58,100	265
Malaysia	1980s [65]	29.7	10,538.1	3.9	166,100	1,056
The Philippines	2016 [66, 67]	98.4	2,765.1	4.6	58,900	103
Taiwan (Chinese Taipei)	1995 [68]	23.4 [69]	20,924.9 [69]	6.6 [70]	99,200	2,902
Thailand	2002 [71]	67.0	5,779.0	3.9	84,500	906
The United Kingdom	1948 [72]	64.1	41,787.5	9.4	53,800	876

## Review

### Do countries committed to UHC implement renal dialysis? Why and how?

Out of the seven study settings, five settings, including the United Kingdom in the 1960s, Hong Kong in 1985 (PD-first policy) [15], Taiwan in 1995 [16], Malaysia in 2001 [17] and Thailand in 2008 [18], have implemented universal access to renal dialysis. The Philippines issued a national policy in 2014 for renal dialysis and implementation is in the early phase. For Indonesia, they have pilot programs and these are under consideration for inclusion in the benefit package. The governments of these five settings (though not all) implemented this program, which is the only life-saving treatment feasible for many reasons. In the United Kingdom, both renal transplantation and dialysis have been provided in the National Health Service since the 1960s. By the end of 2013, 52%, 41.6% and 6.4% of the RRT patients in the United Kingdom received a transplantation, HD and PD, respectively [19]. In Hong Kong and Taiwan, despite the capacity and financial resources to provide renal transplantation, the majority of the population is Chinese and hold a belief against organ donation [20–22]. Thailand and the Philippines do not have enough capacity and financial resources to offer renal transplantation. For instance, the limited supply of donated kidneys [23], lack of infrastructure [10], and shortage of specialized health professionals in the public sector [24] are major barriers to transplantation. Although many studies have demonstrated that renal transplantation is more cost-effective compared to dialysis [25–27], these studies have mostly focused on high-income countries and therefore did not account for costs such as infrastructure development and human resources. Further, research [28, 29] showed that the estimated costs of renal transplantations tend to be higher in the first few years compared to dialysis, which could impact the healthcare budget of developing countries more severely compared to that of developed countries.

In this case, renal dialysis emerges as the most viable option. Apart from being a life-saving treatment, paying for dialysis is well-regarded as a common cause of catastrophic household health expenditure. It was documented that the annual cost of dialysis was 7–48 times higher than the average income of populations in many OECD countries [30]. This is one of the main reasons that many governments decided to include dialysis in their benefit packages.

Among the five countries that introduced access to renal dialysis, each has a different policy regarding dialysis modalities and may be divided into two groups. The first group, Malaysia, Taiwan, and the United Kingdom, offers choices for patients and providers to opt for HD or PD. Patients have no incentive or disincentive to choose the treatment they receive. However, HD is the more frequently chosen dialysis modality, probably due to provider financial incentives as it requires more frequent HD unit visits, resulting in higher professional and HD unit fee earnings. In these three settings, HD accounts for 88% in Malaysia (versus 12% for PD), 91% in Taiwan (versus 9% for PD), and 43% in the United Kingdom (versus 7% for PD) [31–35]. On the contrary, the second group, Hong Kong and Thailand, has instituted a policy on PD as the first-line treatment through the creation of incentives for provider and patient PD use. This includes full reimbursement of PD and only reimburses HD for patients with contraindications for PD. In Thailand, the UHC scheme offers capital investment for PD providers, free training for health professionals and infrastructure development. Moreover, the Thai UHC scheme also allows health professionals to receive a professional fee for providing PD services. As a result, as of 2012, more than 70% of ESRD patients in Hong Kong and 42% of new ESRD patients under the UHC in Thailand now use PD as their treatment [34, 36].

Experiences from Hong Kong [37] and Thailand [38] demonstrate that PD has relatively lower cost for providers (including capital investment), less healthcare provider staff needed, and much lower travel time and cost for patients, leading to increased patient autonomy and satisfaction. This lower travel time and cost for patients reflects the results of a study in South India indicating that direct medical care costs for haemodialysis accounts for only 55% of the total cost, whereas direct non-medical costs, e.g. travel costs, account for around 20% and indirect costs, e.g. opportunity cost loss from work, account for 25% of the total cost [39]. The latter two components make haemodialysis unaffordable by the majority of the population even if dialysis is offered for free. In Thailand, many rural hospitals have successfully implemented PD services for patients who are living in remote areas (e.g. mountain villages) as patients have no need to travel to health facilities often and can administer the treatment themselves at home [40]. Nevertheless, patients experience higher complications such as peritoneal infections due to poor education and those in low socio-economic groups (though they may also have difficulties accessing HD). Caregiver and patient burn-out is also a challenge. HD, on the other hand, has advantages as described by speakers from Malaysia, the Philippines and Taiwan. For example, less patient responsibility and higher provider comfort with the HD process. In the literature, authors found that HD offers better patient socialization as they gather together in the HD units for long periods of time [41].

In Indonesia and the Philippines, where the incidence of ESRD and haemodialysis facilities have gradually been increased simultaneously, RRT is categorized as a high-cost treatment for ESRD. Most patients cannot afford access to the treatment. Therefore, government health insurance recently included RRT in their benefit package, though in practice, the effective coverage is limited. For example, the cost of dialysis is partially supported with certain co-payments that remain unaffordable for the poor [42, 43]. The majority of patients in Asia remain unable to access RRT. It was estimated that the gap between the actual number of patients undergoing RRT and the estimated number of patients with ESRD is very high. For example, in Indonesia, there are around 100,000 people who need RRT but do not have access to the treatment [44].

PD and HD each have their advantages and disadvantages (Table 2) [41, 45, 46]. While PD provides several advantages in terms of clinical outcomes, government treatment implementation, and patient uptake and access, the selection of PD or HD will usually be based on patient motivation, desire, geographic distance from an HD unit, physician and/or nurse bias, patient education and reimbursement policies [47]. Unfortunately, many patients are not educated on PD before beginning dialysis [48]. However, PD can improve patient survival in the first few years, retain residual kidney function, lower infection risk, and increase patient satisfaction while reducing financial stress to governments by addressing the burden of managing the growing number of ESRD patients [49].

**Table 2:** Comparison of advantages and disadvantages of haemodialysis and peritoneal dialysis

	Haemodialysis (in centre, hospital)	Peritoneal dialysis
Advantages	• Patient does not need to be taught to carry out treatment	• Better survival rate within the first 1–2 years
	• Social support system	• Increased patient autonomy
	• Applicable to a majority of patients	• Lower cost
Disadvantages	• Increased time and cost associated with transportation to the hospital	• Patients must be disciplined about maintaining hygiene
	• Increased risk of infection or complications	• Technique failure may lead to infection or complications
		• Potential burnout of patients or caregivers

The governments in Thailand and Hong Kong introduced the PD first policy in order to ensure that PD is promoted over HD based on the reasons stated above. This policy can be seen as a limitation of freedom of patient choice. Nevertheless, it can be argued that government priority is to ensure overall societal benefit, and in these circumstances, the individual benefit may not always be in line with society’s interests and choice may not be available to all [50]. For example, if most patients are given the choice between PD and HD, the budget may be insufficient to cover to the last patient or only patients in urban areas are able to choose between PD and HD, while patients in rural areas may receive only PD.

In conclusion, most settings committed to UHC implemented universal access to renal dialysis because renal dialysis is an essential service; it is life-saving without a better alternative except transplantation, for which efforts should continuously be made in order to make donation and transplantation more widely available. Despite incurring a high cost and not representing good value for money, the governments of these settings consider that the inclusion of renal dialysis is aligned with the objectives of UHC in terms of financial protection. There may be several factors, such as geography and political economy, which affect governments’ decisions to promote either dialysis modality. Experiences from these five settings show that governments contending with the following factors should opt for PD as the first-line treatment: limited UHC budget allocation for dialysis program, less human resources for health, and geographical difficulties in healthcare facility access.

### How to ensure quality of care

Clinical care for ESRD patients should be considered as part of the integrated care for CKD patients. A holistic approach to observe the progression of the disease since the early stage should be taken into account. Healthcare systems should be able to identify populations at risk of CKD and CKD patients in the early stage of the disease in order to provide appropriate care and support and delay disease progression [30]. Healthcare systems should not only focus on investing in clinical care for CKD patients, but there should also be more primary prevention efforts such as promoting a healthy lifestyle (exercise and appropriate diet, e.g. salt and sugar restriction). Experts from all settings had consensus that primary healthcare is an instrumental mechanism as they can reach the majority of the population who are at risk. A study in Bhutan informed that introducing universal access to the Package of Essential Non-communicable disease interventions, including population-based screening and early treatment for diabetes and hypertension, offers very good value for money [51].

All settings that participated in the forum established a RRT registry, which is a database with information on patients undertaking dialysis and/or kidney transplantation. This database can track ESRD patients’ accessibility to RRT by comparing the number of new patients registered to the database to the estimated number of patients based on epidemiological studies; assess quality of RRT services through analysis of survival rates, rate of developing complication(s) or switching between modalities; and patient discontinuation of dialysis treatment. Although the available databases in these settings are at different levels of quality, they should also have information on the socioeconomic status and geographical location of patients in addition to the medical information [52].

In addition, a financial mechanism should be developed to ensure that governments are investing in good quality services and care for renal dialysis. In the United Kingdom, the government developed renal-specific quality indicators, such as blood pressure regulation by angiotensin converting enzyme inhibitors or angiotensin II receptor blocker drugs, and disease progression monitoring by measuring urine albumin and creatinine ratio test [53]. The providers will have incentives to provide this service because they are deemed to be an important part of clinical care. There is strong evidence linking angiotensin converting enzyme inhibitors and angiotensin II receptor blocker drugs with the delay of renal disease progression [54].

In Thailand, palliative care is delivered to ESRD patients who decide to receive palliative care instead of the standard treatment of PD and HD, especially those that are terminally ill or very old. In addition, some patients may have PD and HD for a certain period of time and at some point decide to voluntarily withdraw from treatment. It is important to note that palliative care does not mean lack of care, but it is the provision of medical, physical, and mental support to the patient and their caregiver to ensure that patients at the terminal stage receive humane care [55].

### Improving quality of life of ESRD patients: everybody’s business

CKD has been shown to negatively affect more than just a patient’s physical state of health. It is important to recognize the negative impact that a lifelong or long-term illness has on a person’s quality of life. Depression in patients with renal failure can be accompanied by anxiety in associated partners, two of the most common psychological complications related to the disease. To address CKD patients’ mental health, psychosocial interventions have been put in place by the government in health facilities, healthcare networks, and support centres to improve quality of life. In Thailand, the National Health Security Office has coordinated the creation of networks and volunteer communities for patients with chronic illness since 2003 to provide a space for active volunteers to care for patients. Patient support networks are available for a range of chronic diseases, including renal disease, and these networks work together and collaborate with healthcare professionals to operate a Friendship Support Center in order to provide humanized healthcare [40].

However, the responsibility of ensuring good quality of life for ESRD patients does not lie only with the government – it is also a responsibility of NGOs, the private sector, and communities. Not long after RRT was included in the Thai UHC, Thailand Kidney Friend Clubs were established to extend the Friendship Support networks for CKD patients. The Clubs’ activities include providing information about kidney disease and prevention, visiting homes of PD and HD patients, protecting patient rights, and collaborating with the National Health Security Office and Social Security Office in developing the benefits package. In the Philippines, dialysis solution companies also endeavour to improve patient outcomes and help them cope with illness. For example, these companies also raise awareness on the advantages of PD as a treatment, such as the independence gained from having home-based treatment, which offers a flexible schedule, and maintaining the ability to work and travel. They also work to promote patients’ optimism by organizing activities with partners across Asia such as photo contests, sports competitions, walks, and traveling with a PD train (in which the travellers dialyzed while traveling).

Non-governmental efforts to improve patients’ quality of life are not limited to support groups but also include financial support that helps alleviate the stress of high cost RRT. In the Philippines, the Kidney Foundation of the Philippines, a non-profit organization, runs the Kidney Care Assistance Program, which provides financial aid to patients on dialysis and transplant operation. Fundraising efforts for patients include an annual golf tournament for kidney care, public forums and information campaigns, correspondence to donors and sponsors, media outlets, and kidney care walk-runs.

## Conclusions

This paper addresses the challenges of tackling the increased burden of CKD as part of the UHC policy. Every government’s health ministry or related department(s) should have concrete efforts to minimize increasing incidence and clinical progression of CKD. This can be done not just for CKD, but also overlapping with efforts to deal with other diseases and health risks such as hypertension, diabetes, cardiovascular disease, obesity and unhealthy lifestyles. Since treatment of chronic kidney disease can have catastrophically high costs for patients and their families, the governments should consider programs that can subsidize CKD patients’ costs to a certain extent, depending on available resources and meeting other competing priorities. As the country’s situation changes, governments may revise their policies as appropriate.

Financing, however, is not enough since CKD treatment programs depend on other factors for their success, including human resource development (including more policy-oriented medical education), adequate supply and quality assurance. More research and development for both the clinical and community/psychosocial aspects are needed in order to ensure that the treatment program meets patient needs. This includes creating a patient treatment database. Finally, the government should not be isolated in these efforts; involvement of multiple stakeholders, such as medical associations, industries, civil societies, community groups and networks, and family members, is encouraged.

At the international level, it is important to organize regional forums that bring together not only academics and medical professionals but also decision makers, non-governmental organizations, paramedics and patient group representatives, for sharing experiences, good practices and problem-solving for policy implementation. It is also possible that each country’s national database should follow a common regional platform to share information and conduct monitoring and evaluation across countries. These efforts pave the way for future collaborations between countries, such as regional procurement of dialysis solution for a more affordable and reliable supply as well as joint capacity building activities such as regional PD care training for practitioners, physicians, nurses and healthcare workers.

This study has some limitations. The review is selective and not performed in a systematic manner; however, it is based on priority issues discussed during the international forum. In addition, although the authors tried to address the most important issues on renal dialysis under the UHC program, financial models for funding dialysis were not addressed in depth because since these depend on several factors. Funding dialysis should be in line with the financial model for UHC. Some countries, for example the Philippines, have developed innovative financial models using sin tax to fund dialysis and other high-cost medical care, whereas Thailand pays for UHC and dialysis from general tax.

### Recommendations

1. Every government’s health ministry or related department(s) should implement concrete efforts to ensure a holistic approach for CKD including primary prevention, especially to promote a healthy lifestyle, screening and prompt treatment of diabetes and hypertension. In addition, psychosocial interventions should also be included in the holistic approach for CKD patients in order to improve their quality of life.
2. Since treatment of CKD can have catastrophically high costs for patients and their families, the governments should consider programs that subsidize CKD patients’ costs to a certain extent, depending on available resources and meeting other competing priorities. Financing, however, is not enough because CKD treatment programs need other factors for success such as human resource development (including more policy-oriented medical education), adequate supply and quality assurance. PD First Policy can be considered the policy choice for resource-finite countries committed to UHC.
3. The government should not be isolated in these efforts; involvement of multiple stakeholders, such as medical associations, industries, civil societies, community groups and networks, and family members, is encouraged.
4. Governments and other stakeholders should support research and development for both clinical and community/psychosocial aspects in order to ensure that the treatment program meets patient needs. This includes creating a patient treatment database (e.g. national registry) and community research.
5. Finally, international collaboration should be initiated for sharing experiences, information and good practices, joint capacity building activities, and even multinational procurement of dialysis-related commodities.

## Abbreviations

CKD: Chronic kidney disease
ESRD: End-stage renal disease
HD: Haemodialysis
LMICs: Low- and middle-income countries
PD: Peritoneal dialysis
RRT: Renal replacement therapy
UHC: Universal health coverage
